# Metabiotics: novel idea or natural development of probiotic conception

**DOI:** 10.3402/mehd.v24i0.20399

**Published:** 2013-04-12

**Authors:** Boris A. Shenderov

**Affiliations:** Laboratory of Biology of Bifidobacteria, Gabrichevsky Research Institute of Epidemiology and Microbiology, Moscow, Russia

**Keywords:** probiotics, metabolome, microbial bioactive molecules

## Abstract

Traditionally, probiotics on the base of live microorganisms are considered to be both beneficial and safe. Unfortunately, their effects may have short-term success or are absent or uncertain. Some symbiotic (probiotic) microorganisms with known beneficial health affects may cause opportunistic infections, increase incidence of allergic sensitization and autoimmune disorders, produce microecological imbalance, modify gene expression, transfer antibiotic resistant and virulence genes, cause disorders in epigenome and genome integrity, induce chromosomal DNA damage, and activate signaling pathways associated with cancer and other chronic diseases. The commercially available probiotics should be considered as a first generation means of correcting microecological disorders. Further, their development will include the selection of natural metabiotics and/or working out the synthetic (or semi-synthetic) metabiotics that will be analogies or improved copies of natural bioactives, produced by symbiotic (probiotic) microorganisms. Metabiotics are the structural components of probiotic microorganisms and/or their metabolites and/or signaling molecules with a determined (known) chemical structure that can optimize host-specific physiological functions, regulator, metabolic and/or behavior reactions connected with the activity of host indigenous microbiota. Metabiotics have some advantages because of their exact chemical structure, well dosed, very safe and long shelf-life. Thus, now metabiotics should not consider myth; they are the result of the natural evolution of probiotic conception.

Neither microbial genes nor microbial names cause any harm to the body, but microbial product may do. The presence of very active microbial biological compounds in the gut may have physiological and pathophysiological consequences for the host. Tore Midtvedt (2008)

According to modern scientific doctrine, the human being is a ‘superorganism’, the consortium of numerous *Eukarya, Bacteria, Archaea*, and *Viruses*. Various host indigenous microorganisms should be considered as essential complex extracorporal physiological systems that play a fundamental role in human health and disease. Host microbiota and other functional and metabolic systems, connected with host eukaryotic cells, are working together profitably for the whole organism and for its separated components in the concrete environment conditions. Unfortunately various unfavorable biotic or abiotic factors and stress agents (diet, age, sex, pharmaceutical, even surgical interventions, etc.) can produce microecological disorders resulting in tissue, organ and regulatory systems disturbances that can lead to an increase of a disease development risk (1–5). It means the modulation of microbe/microbe and microbe/host interactions is an extremely important, fundamental, and applied problem in modern biology and medicine. To date, to maintain and restore the gut microbial community, three therapeutic approaches have dominated: probiotics, prebiotics, and synbiotics (4, 6–10). As defined by FAO/WHO, probiotics are non-pathogenic live microorganisms which, when administered in adequate quantities, offer a health benefit to the host (6). Many therapeutics, dietary foods, and additives containing live probiotic microorganisms have been introduced in practice for human health support, acute, chronic, localized, and systemic disease prophylaxis and treatment ranging from acute or chronic diarrhea, intestinal inflammation pathology to allergy, atherosclerosis, and cancer (5, 11). In the last decade, a transplant of the distal gut microbiota (fecal bacteriotherapy) became popular in the treatment of some inflammation intestinal diseases (12, 13). The introduction of genetically engineered probiotics on the basis of recombinant live microorganisms in medical practice has also been actively discussed (4).

## Undesired properties and adverse affects of live probiotic microorganisms

More than 50 years use of probiotics has shown them to be safe and beneficial, but up until now, we have not been able to define the optimal amount of bacteria for probiotic effects; there is no single mechanism of action for all probiotics. Moreover, the beneficial effects of probiotics may have short-term success, be absent or uncertain (3, 7, 14). The latter may be explained by the low concentration of probiotic biologically active substances (bioactives) achieved in target places during the traditional application of live probiotic microorganisms. The number of these microbial bioactives produced might be inadequate to receive desirable specific effects under *in vivo* conditions (4, 7). Besides, various molecules produced in volume by live probiotic cells may interact with different receptors of indigenous microbes and host cells and simultaneously cause both beneficial and negative effects (15). Modern ‘omic’-technologies have revealed the substantial diversity of the gut microbiome between individuals. Only a few microbial phylotypes (species) are shared between different individuals; about 80% of human intestinal microorganisms in the adult gut are individual on the strain level (7, 10, 16–19). The experimental and clinical studies published in recent years have demonstrated that it is very difficult if not impossible to produce industrially adequate probiotics for supporting indigenous microflora at the optimal level by the simple mechanical selection of the separate or set of probiotic microorganism strains. Additional problems in the design of effective probiotics are connected with limited knowledge about the affect mechanisms of such bio-therapeutics to the host gut microbiota, physiology, and metabolism. However, little is known about the molecular mechanisms of probiotic effects (10, 15, 20).

In recent years, our knowledge regarding the safety of probiotics has changed. It is necessary to bear in mind that any detrimental and harmful consequences may only become apparent after extended periods of probiotic use. In reality, some data now show that not all probiotic bacteria are safe even if they belong to *Lactobacillus* or *Bifidobacterium* species having no traditional genes of pathogenicity (21, 22). Medical reports have advised that lactic acid bacteria and even bifidobacteria used as microbial food cultures or probiotics may be rarely associated with human opportunistic infections (infective endocarditis, sepsis, bacteraemia, pneumonia, abdominal abscesses, peritonitis, meningitis, urological infections, rheumatic vascular diseases) especially in patients receiving antibiotic treatment or those severely immune compromised (21–28). There are increased incidences where these bacteria can be responsible for allergic sensitization and autoimmune disorders (23, 29–32). Symbiotic microorganisms (including probiotic strains) sometimes can increase platelet aggregation aggravating hemolytic uremic syndrome (21, 26); some of them may be a source of toxic metabolites (e.g. biogenic amines) (22). Probiotics found on living microbes being introduced into gastro-intestinal or vaginal tracts may cause unintended harm by gaining a competitive advantage and causing an ecological imbalance as a result of altering the microbe biodiversity and metabolic pathways. Because the vast majority of probiotic strains introduced in practice have been selected on the basis of their strong antagonistic activities against disease causative microorganisms, it seemed that many probiotics could suppress the growth and development of human gut and vaginal lactobacilli and other different indigenous microbiota also (33, 34). They may also alter intestinal metabolism due to their microbial enzymatic activities (35). It is necessary also to remember that some intestinal microbial strains can participate in the transformation of some drugs modifying their activity and/or convert the prodrug to the active product (36–38). Unfortunately, there is practically nothing known about interactions of live probiotic microorganisms with drug function *in vitro* and *in vivo*. The situation become more complicated when new strains belonging to *Enterococcus, Streptococcus, Escherichia, Bacillus, Bacteroides*, or other microbial genera are suggested as a potential probiotic including probiotics that consist of multistrains or are constructed on the base of genetically modified microorganisms. There are data that probiotics (*Lactobacillus GG*) inside the intestinal tract can induce the expression of overran additional 400 new genes involved in immune response and inflammation, cell growth and differentiation, apoptosis, cell-to-cell signaling, and cell adhesion resulting in a wider impact on the host's gene expression than thought before the era of macroarray technologies (39). Oral introduction of *E. faecalis* modulates activities of 42 genes in the gut epithelial cells; these genes are involved in the regulation of the cell cycle, cell death, and signaling (40). It has been also shown that during passages through the intestinal tract, some silence genes of probiotic bacteria may also be induced by host cell signals; newly formed bacterial products are poorly characterized both chemically and functionally (39, 41, 42). For example, the induction of 72 probiotic *L. plantarum* WSFS1 genes was demonstrated in these conditions: nine genes were responsible for sugar transport and the production of different enzymes involved in their fermentation; nine genes for the synthesis of amino acids, nucleotides, cofactors, and vitamins; four genes for out cell proteins controlling resistance to specific host factors; and 46 genes for the production of non-identified proteins (42). It means that the probiotic cells inside the gastrointestinal tract may be involved in the interaction with various host-specific networks (e.g. participation in the degradation and production of different nutrients by different metabolic pathways in the small intestine and colon (18, 43), or in the modification of immune response, cell differentiation, cell signaling, adhesion (18, 44); cell proliferation, tissue development, water and ion metabolism, balance of Th1/Th2 cells, etc. (15, 45). Thus, it can be expected that the expression of known or silence microbial and eukaryotic genes may lead to undesirable effects on human health. In recent years, evidence appeared that between lactic acid bacteria and enteric bacteria as well as between different lactic acid bacteria, horizontal gene transfer may take place. Natural gene transfer usually occurs via the uptake of naked DNA (transformation), viruses (transduction), or plasmids (conjugation). It is well known that the spread of antibiotic resistance is a major global health issue (46). Probiotic bacteria can possess acquired antibiotic resistance genes associated with mobile genetic elements (plasmids, transposons) that permit these organisms to transfer this genetic information to strains of the same species, different species, or even different genera including both commensals and pathogens. Such recombination events might not only result in the distribution of undesired genes among intestinal microorganisms but can produce rearrangements in microbial genomes and change gene expression patterns in recipient bacteria. The gene transfer and recombination events associated with probiotics may produce long-term environmental and health negative consequences (21, 22, 26, 46), including chromosome rearrangement and death of recipient cells (47), alteration of the genomic and epigenomic regulation of gene expression, post-translation modification of gene products, host/microbial cross talk result in the change of human metabolic and behavior reactions (20, 48). Recently, it has been shown (on the *E. coli* model) that the DNA methyltransferases represent potential threats to the epigenomic integrity of cell genomes. When the methylation system enters the cell and begins to methylate the host genome, the methylated DNA-specific microbial DNAse senses the epigenetic changes, causing cell death via chromosomal cleavage (49). Traditional virulence traits should not be present in microorganisms used in food fermentation and probiotics (21, 22). Investigations made during the last decade have shown that some human symbiotic microorganisms can produce substances possessing genotoxic affects in intestinal epithelial cell DNA. Therefore, some commensals (including probiotic strain *E. coli* Nissle 1917) possess a set of genes (*pks* island) that are responsible for the production of double-strand breaks in host cell DNA. Bacteria containing the *pks* genes induce in eukaryotic cells a process called megalocytosis, in which the cell body and nucleus become enlarged and mitosis stops. Sometimes 4 hour is enough for the *pks* island carrying bacteria with eukaryotic cells resulting in the increase of DNA double-strand breaks level. A total of 34% of *E. coli* strains isolated from healthy human intestine content had such *pks* islands. A small number of bacterial cells caused minimal DNA damage in eukaryotic cells; in contrast, exposure to 100 or more bacteria per cell has broken the most nuclear DNA. Thus, the breaks in host cell DNA caused by peptide-polyketide genotoxins of some indigenous (probiotic) bacteria could trigger the various cells disorders, including intestinal cancer development (50, 51). Live cells of *Enterococcus faecalis* being inside the intestinal tract release substantial extracellular superoxide, hydroxyl radical, and H_2_O_2_ during carbohydrate fermentation and via autoxidation of membrane demethylmenaquinone. These oxidants can damage DNA and facilitate development of sporadic adenomatous polyps and colorectal cancer (40, 52). Ethanol and its first metabolite acetaldehyde have been recently classified as a class I carcinogen. The acetaldehyde concentration required for a mutagenic DNA effect increases from 100 to 500 µM. Many microbes (including lactic acid bacteria) used as microbial food cultures in fermented food products and in probiotic manufacturing can convert ethanol and/or glucose to carcinogenic acetaldehyde. The acetaldehyde levels formed may exceed the above-mentioned levels markedly. Furthermore, because living probiotic bacteria can colonize the intestinal tract for a long period of time and produce carcinogenic acetaldehyde locally, potentially they can be more dangerous than traditional dairy lactic acid bacteria because of increased total exposure to this bacterial metabolite (53, 54). Some scientists consider that ‘genetic engineering of the human microbiota will enable to endow its members with new desirable functions that treat diseases or promote health’ (10). In spite of the scientific attractiveness of this idea, from a prolonged point of view, the massed introduction of genetic engineering live probiotic microbes in practice may have extremely dangerous ecological and medical consequences even when using such novel platform of microbial cells design as synthetic biology approaches. Thus, the above-mentioned data and discussion allow everybody to come to the major conclusion that the present state of probiotic knowledge is inadequate for reliable ecological and clinical risk assessment of short and long-term negative consequences; they often could be unpredictable. Up to now, we have no sufficient scientific knowledge to support manipulation of the human microecological, immune, and metabolic systems in an exactly predictable manner by the administration of live probiotics (including gene engineering) especially to infants and young children (26, 35). The public should have the right to participate in discussion concerning known and potentially undesired properties for probiotics made on the base of living microorganisms and have imagination regarding the unpredictable consequences of their long-term use.

## Metabiotic concept

Although the history of live probiotic use does not give any exact identified serious concern, recent well-documented events of adverse effects and uncertainty about the level of their risk require new alternative approaches in prophylaxis and treatment of pathological conditions associated with the imbalance of host microbiota. These approaches have to retain and improve the positive accumulated experience of work with live commensal microorganisms (probiotics) and decrease a safety concern.

Investigations of the last 10–20 years have demonstrated that gut microorganisms (including probiotic strains) are able to break down and metabolize complex food nutrients and endogenous substances (saliva, gastro-intestinal juices compounds, epithelial cells, dead microbial cells, etc.) resulting in the formation of low molecular weight (LMW) bioactives that may be localized both inside and/or out of microbial cells and found in the intestinal content or passing across the intestinal epithelium barrier determined in the various human fluids, organs and tissues. These compounds derived from probiotic (symbiotic) microbes form what has been called the probiotic metabolome. Interacting with corresponding prokaryotic and eukaryotic cell targets, these biological and pharmacological active compounds may control many genetic, epigenetic, and physiological functions; biochemical and behavior reactions; and intra- and intercell exchange of information. Some commensal microbes including probiotics can secrete a variety of signaling molecules that can modify the inter-bacterial signaling (quorum quenching) and suppress the expression of virulence genes in pathogens or stimulate the growth of beneficial indigenous gut microorganisms (15, 18, 20, 48, 55–62). In our opinion, the probiotics commercially available now should be considered as a first generation of means directed for correction of microecological disorders. Future development of traditional probiotics will include improvements of this generation by means of the production of natural metabiotics (manufactured on the base of current probiotic strains) and synthetic (or semi-synthetic) metabiotics that will be analogies or improved copies of natural bioactives produced symbiotic microorganisms. The terms ‘metabiotics’ (4, 20, 63, 64), ‘metabolic probiotics’ (65, 66), ‘postbiotics’ (1), ‘biological drugs’ (10), or ‘pharmacobiotics’ (15) mean small molecules that are the structural components of probiotic (symbiotic) microorganisms and/or their metabolites and/or signaling molecules with determined (known) chemical structure that can affect the microbiome and/or human metabolic and signaling pathways optimizing the indigenous microbiota composition and function and host-specific physiology, immunity, neuro-hormonbiology, regulator, and metabolic and/or behavior reactions connected with the activity of host indigenous microbiota. Different probiotic strains can become the source of hundred (thousands) of LMW bioactives (bacteriocins and other antimicrobial molecules, short chain fatty acids, various other fatty and organic acids, biosurfactants, polysaccharides, peptidoglycans, teichoic acids, lipo- and glycoproteins, vitamins, antioxidants, nucleic acids, different proteins including enzymes and lectines, peptides with various activities, amino acids, growth and coagulation factors, defensin-like molecules or their inductors in human cells, messenger (signal) molecules, plasmalogens, various co-factors, and so on) (2, 7, 10, 15, 18, 20, 48, 55, 56, 67). Various symbiotic (probiotic) strains can produce different sets of such LMW bioactive molecules, attractive candidates for metabiotics construction (Table 1). It should remember that the spectrum and number of bioactives of microbial origin determined in the human different biological fluids and eukaryotic cells may be also connected with the activity of the host transmembrane transporters and/or liver and other tissue enzymes that can carry or transform various microbial substances. Metabolomics analysis of plasma extracts from germ-free and conventional mice (68) as well as other hosts and gut-microbial co-metabolomes revealed a significant interplay between gut microflora and mammalian metabolism (37).

**Table 1:** Some groups of LMW compounds of symbiotic (probiotic) microbe origin that may become the basis for manufacture of potential metabiotics

Bacteriocins
Short-chain fatty acids, other organic acids
Proteins, peptides, amino acids
Nucleic acids, nucleotides
Polysaccharides, peptidoglycans, other cell surface molecules
Plasmalogens, vitamins, antioxidants, co-factors
Various messenger (signal) molecules

Specific representatives of these groups of LMW compounds isolated from symbiotic (probiotic) microorganisms or their cultural liquids may be used for manufacturing microbe-free food supplements, functional foods, and drugs for prophylaxis and treatment of chronic human diseases, as well as sport and anti-aging foods, etc. The above -mentioned approaches and tools for the design of new types of bio-actives on the base of LMW molecules of symbiotic microbiota origin for nutrition and medicine are already being developed in some countries (60).

Current probiotic strains in such situations may become starter strains for industry manufacturing of such microbial bioactive molecules. The knowledge of quality and quantity of LMW molecule profile of each industry used or potential probiotic strain will help researchers to design novel metabiotics with increased health effectiveness, and determine the optimal frequency, dose, and mode of their administration. Industrial production of metabiotics could become the novel prophylaxis and therapeutic approach to address human health in the near future because of their potential ability to interfere in the processes associated with stability of host genome and microbiome; modulation of epigenomic regulation of gene phenotypic expression in eukaryotic cells; improvement of the information exchange, signaling and metabolic pathways in numerous bacteria, bacteria–host and host systems that play an important role in the control for many genetic, epigenetic, physiological functions, biochemical and behavior reactions; in cell growth and host development; in supporting host health in general. We should always remember that real health effectiveness of suggested metabiotics depends on our knowledge of the physicochemical characteristics of metabiotic molecules (molecule isomerism: l or d, and α, β or γ forms; valency and isotope state of incoming chemical elements; substance solubility; dispersiveness, ligand binding, oxido-reduction potential of metabiotic ingredient(s), its(their) interaction with other components that can be both as enhancers or inhibitors; competitive inhibitors for specific transport proteins or absorption site) as well as host physiological status, illness, constituents of the diet, medication, and so on.

The introduction of the concept ‘Metabiotic’ in practice permits including in biotechnology not only bifidobacteria, lactobacilli, *Escherichia*, enterococci strains but also tens and hundreds of other strains belonging to human dominant intestinal phyla (*Bacteroides, Firmicutes, Proteobacteria, Actinobacteria*, and *Archae*) for nutrition and medical aims. There is no doubt that the future metabiotics created on the base of strains belonging to the widen spectrum dominant microorganisms may be more effective at conferring health benefits than the LMW bioactives received only on the basis of classical probiotic strains (bifidobacteria and lactobacilli). The known and potential microbial starter cultures of human microbiota origin that could be used as a platform for metabiotic production should firstly be investigated on the synthesis of structural and excreted bioactive substances participating or regulating in pathways associated with carbohydrate, fat, and cholesterol metabolism; work of immune, hormone, and nervous systems; metagenome and epigenome stability; and gene expression regulation. Undoubtedly among human gut microbiota there are species and strains which are potential sources of small diffusible antimicrobial agents, regulators of host energy balance, cholesterol-lowering compounds, modifiers of immune reactions, stimulants, antidepressants, cognitive enhancers, etc. (10, 60, 63). Development of databases of microbe-derived individual compounds or groups of compounds provides key information, which could be used toward goal-directed design metabiotics with specific medical effects. It is also important to remember the knowledge that widen rosters of LMW microbial metabolites and signal molecules produced by microorganisms and determined in the human physiological fluids can have the great diagnostic value (2, 37, 69, 70). Because *in vivo* production of bioactive small molecules of human and microbial origin is often connected with prebiotic-stimulated secondary metabolism (8, 10), there is a sense to design combined metabiotic/prebiotic foods and drugs for nutritional and medical purpose and prescription targeted to host microbiota or to indigenous microflora associated host functions, metabolic and behavior reactions.

## Known and potential metabiotics

Some metabiotics on the base of natural (or artificial) bioactives of microbial origin in the last decade have already been introduced into medical practice and have proven their effectiveness in reducing infectious and metabolic diseases. For example, nowadays on the international market of drugs there is the most known metabiotic with trade mark ‘*Hylak Forte*’ (Ratiopharm/Merckle, Germany. Code EAN: 4030096245166; N P No1497/01, 2009-05-14). This bacteria-free liquid medicine for oral use contains metabolic products of *Escherichia coli* DSM 4087 (25 g), *Streptococcus faecalis DSM 4086* (12.5 g), *Lactobacillus acidophilus DSM 4149* (12.5 g), and *L. helveticus DSM 4183* (50 g). The benefits of this sterile liquid drug are explained by the presence of SCFA, lactic acid, and some other non-identified microbial metabolites in this drug. There are data that ‘*Hylak Forte*’ promotes the health of adults and children thanks to producing positive shifts in intestine microbiota, host acid-alkaline, water–salt metabolism, vitamins B and K balance, energy provision to intestinal epithelia and local immune cells (71). Among other commercial metabiotics, it may also be named ‘Zakofalk’ (a combination of sodium butyrate and inulin) recommended for the treatment of mild to moderately active inflammation intestinal diseases (72); *E. coli* glycoprotein with anorexic activity (73); *Lactobacillus casei* (LEx) polysaccharide – glycopeptide with antihypertension effect (74, 75); *L. helveticus* three-peptides (Val-Pro-Pro and Ile-Pro-Pro) inhibiting angeotensin convert enzyme (ACE) and resulting in the decrease of blood pressure (76, 77); lyophilized *B. subtilis* bacteria-free cultural fluid containing lysozyme, catalase, polypeptides, peptidoglycan, amino acids, and natural sorbent ceolite with immune-modulator and gut microbiota-restoring activity (“bactistatin”) (78; www.stada.ru/products/baktistatin.html); lactic acid bacteria SCFA and other organic acids (‘Solcarmon’, ‘Frodo’) (79); Quorum sensing *Escherichia*, lactobacilli and bifidobacteria autoregulators of growth and development of indigenous intestinal microflora (65, 66). Potential metabiotics on the basis of lactobacilli, bifidobacteria, enterococci proteins, peptides, adhesions (55, 56), biosurfactants (more than 30kD) (80), lectines (81, 82), nucleic acids and various cell wall molecules (15) connected with microbial cells or produced in cultural liquid possessing various host effects are now under development. Special attention to my mind it should be paying to microbial membrane sterol-like compounds (e.g. plasmalogens) (59) and outer membrane lipoproteins (e.g. lipocalins) (83, 84) as attractive candidate molecules for the manufacture of different types of metabiotics that are capable of inhibiting lipid and energy metabolism, immune, hormone and nervous system functions.

Metabiotics, as modifiers of physiological functions, biochemical and behavior reactions, have some advantages. They have exact chemical structure, well dosed, very safe and long shelf-life. Besides, metabiotics possess the better absorption, metabolism, distribution, and excretion abilities compared with classic probiotics on the base of live microorganisms. A detailed molecular understanding can permit metabiotics to become significant specific and active contributors to the benefits derived from probiotics (4, 10, 15, 20). If the producer tries to label the metabiotic product with the specific therapeutic claims (prevent or treat a specific disease or improve the specific function or metabolic reaction), such metabiotics must be regulated as a drug needing approval by separate Regulatory Acts of corresponding National and International Agencies controlling Quality and Safety of Foods and Drugs.

## Conclusion

I now consider metabiotics not to be a myth; they are a natural evolution of the probiotic conception. In the near future on the base of probiotic LMW bioactives with proven specific benefit effect(s), a set of semi- and/or completely synthetic metabiotics will be designed that are analogies or improved copies of the natural microbial bioactives. This is a similar route of development to the traditional antibiotics that have been coming out during the last 50 years. LMW bioactives of indigenous microbiota origin being even chemically similar with the molecules of other origin (e.g. from food raw materials or food-stuffs) have advantages in principle. For many millions years of evolution, a human superorganism has been selecting prokaryotic and eukaryotic microorganisms from the environment that were functionally and metabolically the most optimal for human life and development. It means that metabiotics manufactured on the base of LMW bioactives of indigenous microbiota origin are the most beneficial and the safest.

The metabiotics of next generations will promote the further development of probiotic concept, improve effectiveness and benefit specificity of classic probiotics, and reduce environmental and health hazards of the current microecological approaches in the prevention and treatment of diseases associated with the imbalance of host microbiota.
